# Supplementary figures and images for: Semaphorin7A patterns neural circuitry in the lateral line of the zebrafish
Source: eLife. 2024 Aug 12;12:RP89926. doi: 10.7554/eLife.89926 (PMC11318972; doi:10.7554/eLife.89926)

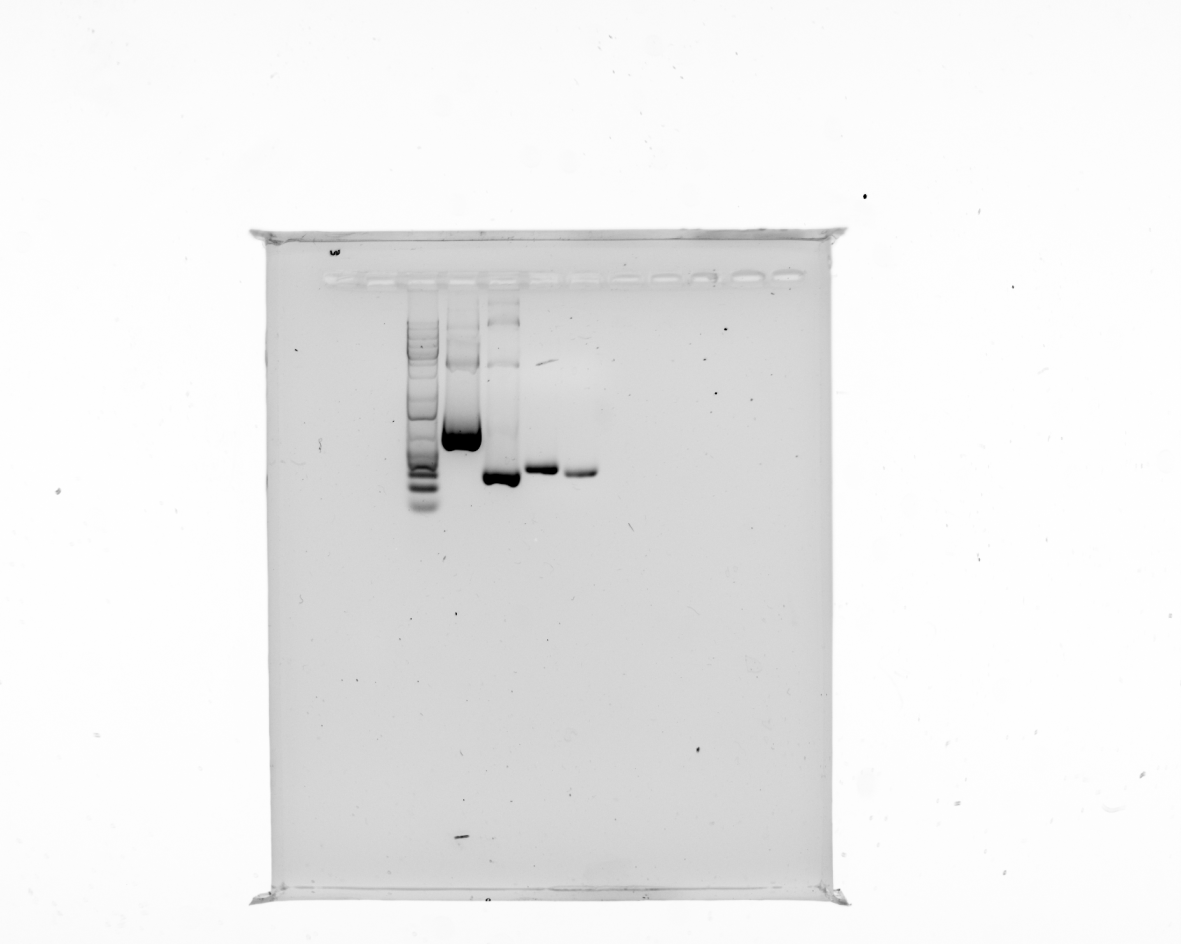

Supplement: Figure 1—source data 1. [file elife-89926-fig1-data1.zip › GPI_Sec_pvalb8_s100t (1).tif]

# The full raw unedited gel

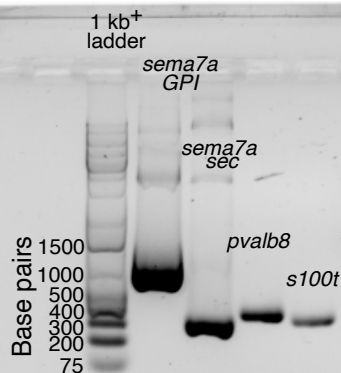

# Cropped gel image

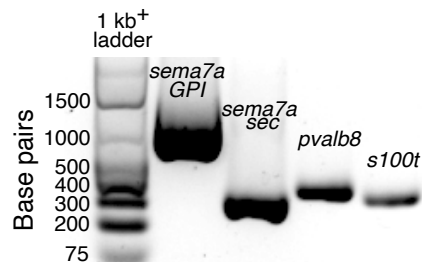

Supplement: Figure 1—source data 1. [file elife-89926-fig1-data1.zip › labelled raw unedited gel.pdf]

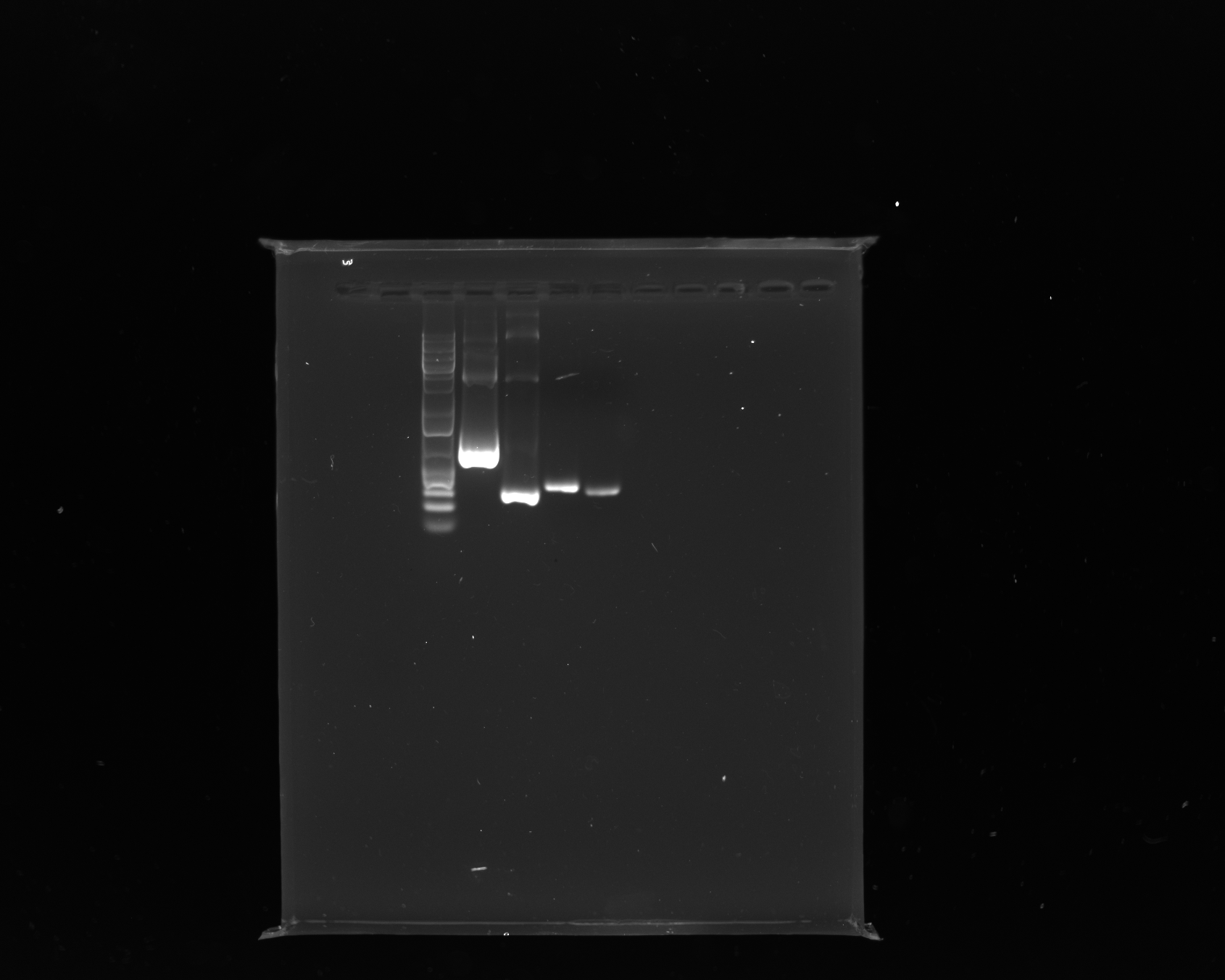

Supplement: Figure 1—source data 1. [file elife-89926-fig1-data1.zip › NUCLEIC_ACID_01112024_151443_(Nucleic Acid)_raw (1).tif]
